# Infection with *Pythium flevoense* in a harbour porpoise (*Phocoena phocoena*) as a novel cause of dermatitis in marine mammals

**DOI:** 10.1186/s13567-023-01226-1

**Published:** 2023-11-02

**Authors:** Edwin J. B. Veldhuis Kroeze, Cornelis E. van Elk, Marco W. G. van de Bildt, Peter R. W. A. van Run, Geoffrey Foster, Nissrine Abou-Chakra, Rasmus Krøger Hare, Thijs Kuiken

**Affiliations:** 1 Department of Viroscience, Erasmus University Medical Centre, Rotterdam, The Netherlands; 2 SRUC Veterinary Services, Inverness, IV2 5NA UK; 3 Unit for Mycology, Department of Bacteria Parasites and Fungi, Statens Serum Institut, Copenhagen, Denmark

**Keywords:** Novel pathogen, oomycete, *Pythium flevoense*, marine mammal, cetacean, harbour porpoise, dermatitis

## Abstract

The oomycete *Pythium flevoense* was diagnosed as the cause of dermatitis in a young adult female harbour porpoise (*Phocoena phocoena*) that had been trapped in a pound net in a temperate saltwater environment. Disease from *Pythium* sp. infection—pythiosis—is infrequently diagnosed in humans, horses, dogs, cattle, and few other mammalian species. Pythiosis is typically associated with exposure to tropical or subtropical freshwater conditions, and typically caused by *Pythium insidiosum*. However, until now, pythiosis has been reported in neither marine mammals nor temperate saltwater conditions, and *P. flevoense* is not known as a cause of pythiosis in mammals. This porpoise developed generalised dermatitis despite treatment and euthanasia was necessary. Histopathological evaluation revealed a chronic active erosive dermatitis, with intralesional hyphae morphologically consistent with a *Pythium* sp. PCR analysis and sequencing of affected skin matched *Pythium flevoense* with a 100% similarity to the reference strain. Additional diagnostics excluded other pathogens. Based on this case report, *P. flevoense* needs to be considered as a mammalian pathogen. Furthermore, harbour porpoises and possibly other marine mammals may be at risk of infection with *P. flevoense*, and pythiosis should be included in the differential diagnosis of dermatitis in marine mammals.

**Supplementary Information:**

The online version contains supplementary material available at 10.1186/s13567-023-01226-1.

## Introduction

*Pythium* species are oomycetes, fungus-like filamentous eukaryotic organisms belonging to the kingdom Stramenopila, phylum Oomycota, class Oomycetes, order Pythiales, family Pythiaceae, which may reproduce both sexually and asexually [1–3]. Most oomycetes are saprophytic and may cause devastating crop diseases, such as potato blight [4]. However, opportunistic infections with *Pythium insidiosum* in humans and other animals may cause disease, called pythiosis (insidiosi), typically characterized by necrotising and granulomatous inflammation [2, 3]. The incidence and host range of pythiosis seem to be increasing [2, 3], supported by an increase of pythiosis case reports in the last decade [5].

Infection with *P. insidiosum* occurs typically in (sub)tropical stagnant freshwater containing motile zoospores that enter skin lesions and invade tissues. Zoospores show tropism for skin and hair [2]. Pythiosis has been reported particularly in humans, horses, cattle, and dogs, in Brazil, USA, Haiti, Australia, New Zealand, Malaysia, India, and Thailand [5–8]. Pythiosis is not contagious amongst humans and other animals or known to cause zoonotic transmissions [2, 3].

Depending on the site of infection and involved species, cutaneous, lymphonodular, osseous, vascular, ocular, visceral, and disseminated forms of pythiosis may occur [3]. Vascular pythiosis is reported in humans, typically exposed barefooted to water in rice fields in Thailand. Haemoglobinopathies, anaemia and leukaemia are risk factors for infection in humans [9–11]. In general, immunosuppressed patients may have increased risk of disease [12]. Pythiosis in horses manifests usually as ulcerative necrotising granulomatous dermatitis containing horse-specific central coral-like calcareous cores or “kunkers”, on limbs and ventral body [3]. Pregnant horses may have increased risk of disease [13]. The classical diagnosis of pythiosis may be laborious and time-consuming as it relies on culture identification [14] or immunohistochemistry [15]. Faster clinical serological diagnostic tests [16–18], and molecular diagnostic techniques by DNA extraction from infected tissue with PCR-based assays and internal transcribed spacer (ITS) sequence analysis [11, 19, 20] are now available.

Histologically, *P. insidiosum* is morphologically discernible as hyaline, pauci-septate, nonparallel thin-walled (5–10 μm) hyphae (also often more appropriately referred to as pseudohyphae since it concerns oomycetes thus not being true fungal hyphae) with infrequent and irregular branching. As routine H&E stains may fail to stain these hyphae, enhancement of discernibility requires silver stains like Grocott silver stain [3, 15].

*Pythium flevoense* was isolated and identified for the first time in soil samples from the province Flevoland, The Netherlands in 1968 [21]. Sporadically it is reported to infect crustacean copepods (*Parabroteas sarci*) in Argentina [22] and ayu fish larvae (*Plecoglossus altivelis*) in Japan [23]. Here we present the clinical, pathological, molecular, and microbiological findings of the first case of mammalian disease caused by *P. flevoense*, and simultaneously the first case of pythiosis in a marine mammal, a harbour porpoise (*Phocoena phocoena*).

## Materials and methods

### Harbour porpoise

A female harbour porpoise, estimated to be 14 months of age, was found alive on the 22^nd^ of May 2018, trapped in a pound net in the coastal seawaters near the town Korsør in Denmark. It was freed and brought to the Marine Biological Research Center, University of Southern Denmark, and intended to be part of the porpoise collection at the neighbouring sea aquarium Fjord & Bælt at Kerteminde Bay which is permitted by Danish law to house bycaught harbour porpoises for research and conservation purposes. It was named Idun and kept alone in a quarantined saltwater basin. A separated basin housed another normal healthy harbour porpoise. Both basins were circulated continuously with the same fresh seawater from the bay. Despite treatments for a progressive generalised erosive dermatitis, the animal did not recover and was euthanised after 3½ months (details in Results; clinical history). The carcass was stored on wet ice directly after euthanasia until autopsy was performed, 24 h after death.

### Pathology and histology

During autopsy the porpoise’s external and internal organs were examined. The skin lesions were photographed and sampled for histology, bacteriology and molecular analysis. Internal organs and tissues were similarly sampled. Samples for histology of skin, lung, trachea, heart, kidney, spleen, lymph nodes, urinary bladder, liver, pancreas, stomach, small and large intestines were fixed by immersion in 10% neutral-buffered formalin. These fixed samples were routinely processed and embedded in paraffin. Tissue sections of 4 μm thick were mounted on glass slides, deparaffinised with xylene, rehydrated using graded alcohols, and stained according to routine histochemical protocols [24] with haematoxylin and eosin (H&E) and with Grocott silver stain for histopathological examination by light microscopy. The H&E sections were evaluated for any histopathological changes.

### Molecular and microbial analyses

Samples from affected skin were biopsied from the live animal on day 34 after rescue and taken during autopsy on day 105 after rescue. Samples for molecular analyses were stored in lysis buffer until processing. Tissue samples for bacteriology including affected skin were taken during autopsy on day 105, stored and frozen at −20 °C and subsequently thawed a week later for further analyses. Fluorescent microscopy using Blancophor was performed on the skin samples (taken on day 34), to visualize fungal(-like) organisms. Fungal cultures from the same samples were performed using SGA agar (sabouraud glucose agar; SSI Diagnostika or bioMérieux) and YGC agar (yeast glucose agar; SSI Diagnostika) with incubation at 35–37 °C for 2 weeks. Species identification included classical techniques including macro- and micromorphology and thermotolerance testing. Molecular analysis included internal transcribed spacer (ITS)-PCR and sequencing of which the results were compared with online sequence databases in July 2018, NCBI (BLAST) and CBS* database. (*Centraalbureau voor Schimmelcultures, The Netherlands; current name: The Westerdijk Fungal Biodiversity Institute, Utrecht, The Netherlands). The sequence analysis was repeated in August 2021 specifically for this study.

For bacteriological examination, samples of skin, lung, kidney, liver, spleen, uterus, tracheobronchial lymph node and adrenal gland were frozen at −20 °C and after thawing, cultured according to an in-house established standard protocol, including classical methods and commercial API identification kits (bioMérieux, Basingstoke, UK), for bacterial species from marine mammals, as described previously [25]. For virological molecular analysis tissue samples of brain, lung, kidney and urinary bladder collected during autopsy were tested by a pan Paramyxovirus reverse transcriptase polymerase chain reaction (RT-PCR) [26]. Samples stored in RNA later were washed with PBS, homogenized, and RNA was isolated using the High Pure RNA Isolation Kit (Roche Diagnostics GmbH, Mannheim, Germany), following the manufacturer’s protocol. After first strand synthesis using Random Primers (Promega Benelux), pan Paramyxovirus primers PAR-F1: 5′GAAGGITATTGTCAIAARNTNTGGAC3′ and PAR-R: 5′GCTGAAGTTACIGGITCICCDATRTTN C3′ were used for the first PCR followed by primers PAR-F2: 5′GTTGCTTCAATGGTTCARGGNGAYAA3′ and PAR-R: 5′GCTGAAGTTACIGGITCICCDATRTTNC3′ for a seminested PCR. The resulting PCR reactions were assessed on 2% agarose gels.

## Results

### Clinical history

Upon accession on the 22^nd^ of May 2018 into the Marine centre (day 0), blood samples were collected. Results from standard haematological and clinical biochemical analyses on these samples were all within normal ranges, without any indication of immunosuppression, anaemia, or inflammation (see Additional file 1 for the results of all blood analyses including reference intervals as previously published [27]). The animal was in a normal healthy condition without skin lesions upon accession. From day 0 onwards, regular dyspnoea started to develop, characterised by coughing and intermittent forceful expirations, so-called “jet” breaths. Initial breathing frequencies were within normal range of 15–20 times/5 min, but started to rise from day 15 onwards to 20–32 times/5 min. On day 102 breathing frequency suddenly increased to 45 times/5 min. Coinciding with this increase, lethargy, listing (to the left), and failing to dive were observed. The animal appeared like an inflated balloon, resurfacing when pressed underwater.

From day 9 onwards, small white spots were first noticed in the skin of the head and flank, and quickly developed into enlarging brown spots that started to erode over the following days. Skin biopsies obtained on day 22 were examined by light microscopy and revealed inflammation and a fungus-like infection of the epidermis. PCR analysis and genetic sequencing of skin samples taken on day 34 identified *P. flevoense* DNA. The degree and severity of the dermatitis progressed rapidly despite several antifungal treatments. The progression of the disease and its unresponsiveness to therapy, in combination with debilitating clinical status and deteriorating blood values (decrease in haematocrit [Ht] from 45.8 to 31.2%; increase in white blood cells [WBC] from 5.88 to 28.22 × 10^9^/L) (Additional file 1), and poor prognosis led to euthanasia on day 104.

The various medicinal treatments applied were: days 0 to 3 and days 10 to 29: amoxycillin with clavulanic acid (220/55 mg oral b.i.d. [twice a day]), days 3 to 29: enrofloxacin (150 mg oral b.i.d.), days 20 to 104: tramadol (20 mg oral b.i.d.), days 25 to 31: itraconazole (100 mg oral b.i.d.), days 27 to 37: miconazol (20 mg/g gel topical q.d. [once a day]), days 29 to 49: terbinafine (60 mg oral q.d.), days 31 to 45: posaconazole (150 mg oral b.i.d.), days 45 to 101: voriconazole (120 mg oral q.d.), days 45 to 104: amoxycillin with clavulanic acid (250/62.5 mg oral b.i.d.), days 100 to 102: posaconazole (300 mg oral q.d.), days 100 to 103: terbinafine (60 mg oral q.d.), days 101 to 103: enilconazole (14.4% w/w baths), days 103 to 104: enrofloxacin (150 mg oral b.i.d.).

### Pathology

**Figure 1:**
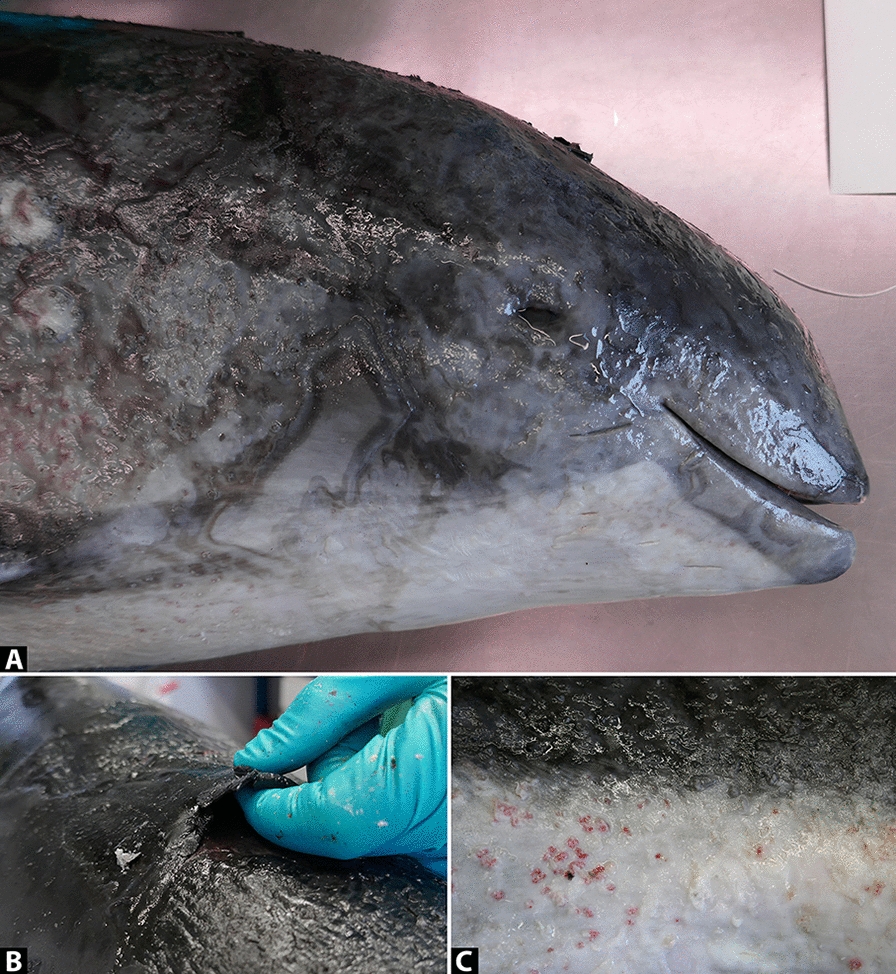
**Macroscopy of cutaneous pythiosis in a harbour porpoise.** **A** Post-mortem photograph of the harbour porpoise’s head with cutaneous pythiosis characterised by extensive multifocal to coalescing erosive dermatitis. **B** Close-up photographs exhibiting a partially detached necrotising skin flap of the dorsum, and **C** multiple small red erosions of the skin of the tail.

Macroscopic examination showed a female non-pregnant harbour porpoise in moderate physical condition (body weight: 25.9 kg, body length: 116 cm, axillary girth: 77 cm, and blubber thickness in front of dorsal fin: 17 mm), with a generalised dermatitis. The dermatitis was characterised by multifocal to coalescing lesions with an irregular rough surface (Figure 1A) occupying approximately 70% of the body surface. The centres of these lesions consisted of many irregular red erosions with a range of 1–3 mm in diameter (Figure 1C) surrounded by a darker coloured inner rim of 3 mm width and an adjacent paler coloured outer rim of 5 mm width. The lesions were depressed below the normal skin surface by 1 mm. The hypodermis appeared unaffected on cut section. An inflamed skin flap of 70 × 80 mm had partially detached between the dorsal fin and the blowhole (Figure 1B). Underneath this skin flap normal healthy skin was present. On the left side of the body, three well-delineated white foci of 3–4 mm diameter of intact skin were present, consistent with old scars.

Upon opening the thorax, the right pleural cavity contained air instead of a normal vacuum, consistent with a unilateral pneumothorax. This finding explained the difficulty in diving and listing to the left. The prescapular, para-cervical, lung-associated and dorso-abdominal lymph nodes were markedly enlarged and bulged on cut surface with a yellow uniform appearance. The pulmonary lymph nodes were yellow-red mottled on cut surface. The liver was enlarged, pale with a red and yellow lobular pattern and had rounded edges. No further significant abnormalities were detected at autopsy.

**Figure 2:**
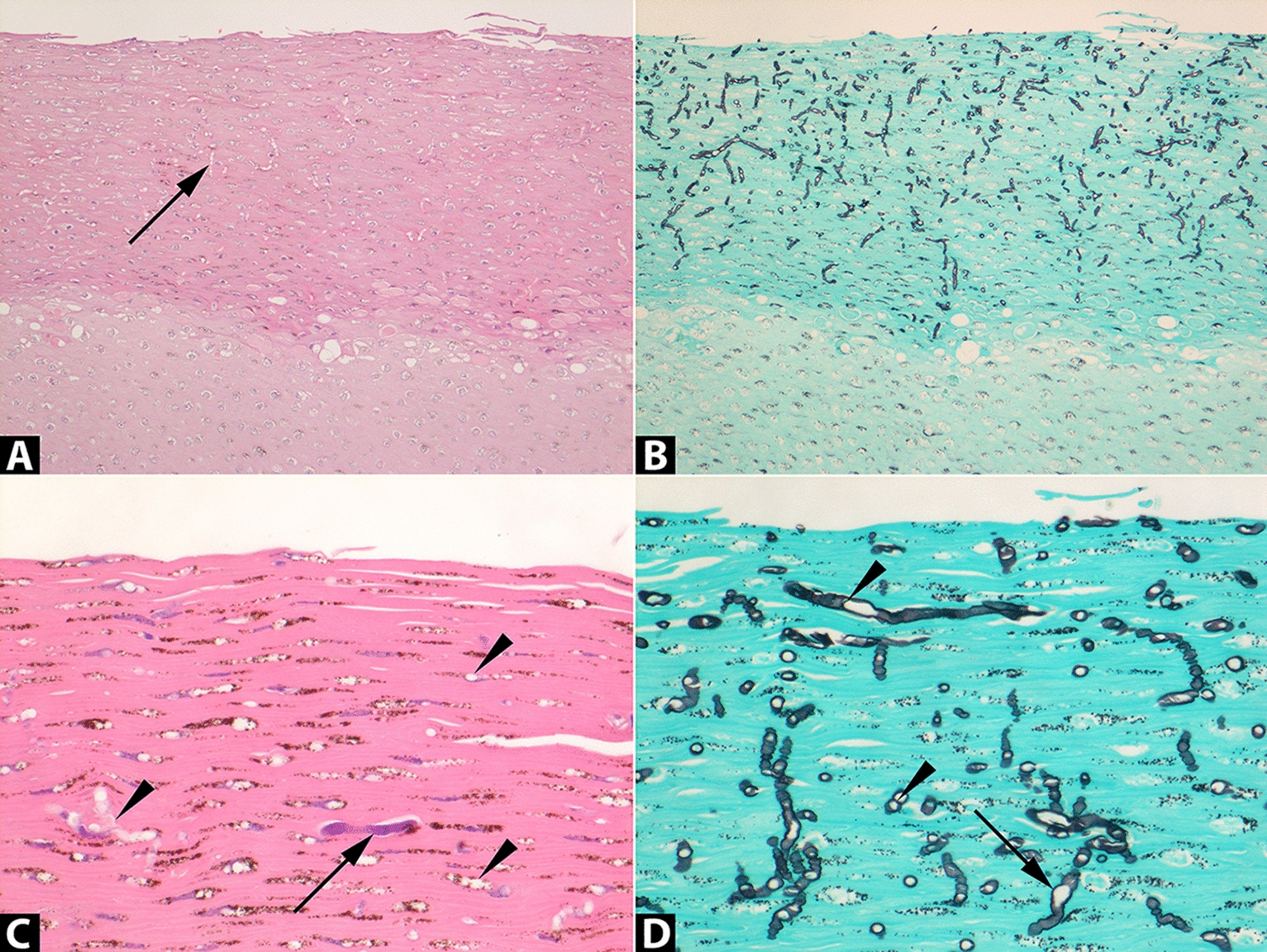
**Histopathology of cutaneous pythiosis in a harbour porpoise.** Sections of skin stained with Haematoxylin & Eosin (H&E; **A**, **C**) or Grocott silver stain (**B**, **D**). Original magnifications: ×100 (**A**, **B**) or ×400 (**C**, **D**). **A** The *Pythium flevoense* organisms predominantly invaded the superficial layer of squamous non-keratinised epithelium of the skin (darker stained top layer) and were poorly visible in the H&E-stained tissue as well-delimited round to oval empty spaces (arrow) compared to **B** the high visibility in the Grocott silver stain showing the abundant presence of black-stained organisms. **C** Close-up of a rare H&E-stained hypha (arrow) amidst many non-stained hyphae (arrow heads) compared to the Grocott silver-stained serial section **D** showing many irregular angular and infrequent septate (arrow heads) hyphae with frequent bulbous swellings (arrow).

On microscopic examination of affected skin, the superficial layer of squamous non-keratinised epithelium of the epidermis was thickened due to hyperplasia and contained fungus-like hyphae occasionally seen in routine H&E stains that were in fact numerous and readily discernible in Grocott silver stains (Figure 2). The pauci-septated hyphae, mostly perpendicularly orientated in relation to the skin surface, measured approximately 7–10 μm in diameter with frequent bulbous swellings and rare irregular branching. Neutrophils infiltrated into the epidermis and formed several micro-abscesses and serocellular pustules, some of which contained hyphae. In few skin samples hyphae were encountered also within the deeper layers of the epidermis. Furthermore, mild to moderate mixed inflammatory cellular infiltrates and proliferation of capillaries were present within the superficial dermal papillae. This infiltrate was composed of mainly neutrophils admixed with fewer lymphocytes. No bacteria, fungi, protozoa, or viral inclusion bodies were observed in the inflamed skin. These findings are consistent with a diagnosis of a chronic active erosive dermatitis with intralesional fungus-like organisms.

Sections from the right lung were affected by a mild to moderate chronic-active multifocal necro-suppurative bronchopneumonia with intralesional nematodes and bacteria. The prescapular, para-cervical, lung-associated and dorso-abdominal lymph nodes showed moderate chronic reactive lymphoid hyperplasia in conjunction with the presence of mild to moderate numbers of neutrophils and eosinophils within the sinuses. The liver showed mild acute multifocal centrilobular hepatic necrosis.

### Genetics and microbiology

When performing fluorescent microscopy using Blancophor on skin samples (taken on day 34), threadlike structures were discernible. These structures did not resemble true hyphae. They lacked the usual characteristics of fungal hyphae since they were of significantly variable wideness, had no distinct regular septa, and the tips were cut rather than rounded hyphal ends and fluorescence was not distinct as is usual for fungal hyphae.

After 2 weeks, fungal culture of one of four skin samples (taken on day 34) grew *Aspergillus niger* on YGC agar only. As *A. niger* is found ubiquitously as cause of black mold on certain fruits and vegetables, this single culture growth is considered more likely as a contaminant rather than a primary pathogen.

Specific fungal PCRs on four skin samples (taken on day 34) for *Mucorales* sp. (zygomycetes) and for *Aspergillus* sp. were all negative (likely confirming contaminated growth of *Aspergillus niger* in culture), but all four samples were positive for *Pythium* sp. The results of ITS sequencing in 2018 matched *Pythium pectinolyticum* with a similarity of > 99.5% compared with the reference strain CBS* 122643 (*Centraalbureau voor Schimmelcultures, the Netherlands; current name: The Westerdijk Fungal Biodiversity Institute, Utrecht, the Netherlands). Sequence analysis was rerun in August 2021 for the current study, and this time showed a 100% sequence similarity to CBS strain 234.72 of *Pythium flevoense*, which had been submitted to the database after 2018, and thus the aetiological species was reclassified as *P. flevoense* in this study. The fasta of the sequenced porpoise skin samples were:

>ITS1&2GCCCGTCGCACCTACCGATTGAATGACTCGGTGAGAAATCGGGACCGTGAATCCGTTTGCTTCATTGCGAGTGGACTTATGGGAACTTTTTCTAACCTCGCCATTTAGAGGAAGGTGAAGTCGTAACAAGGTTTCCGTAGGTGAACCTGCGGAAGGATCATTACCACACCCTAAAACTTTCCACGTGAACCGTTGTAAATATGTTCTGTGCTCTCTCTCGGGAGAGCTGAACGAAGGTGGCCTGCTTAATTGTAGACTGCCGATGTACTTTTAAACCCATTAAACTAATACTGAACTATACTCCGAAAACGAAAGTCTTTGGTTTTAATCAATAACAACTTTCAGCAGTGGATGTCTAGGCTCGCACATCGATGAAGAACGCTGCGAACTGCGATACGTAATGCGAATTGCAGAATTCAGTGAGTCATCGAAATTTTGAACGCACATTGCACTTTCGGGTTATGCCTGGAAGTATGCCTGTATCAGTGTCCGTACATCAAACTTGCCTTTCTTTTTTTGTGTAGTCAAGATTAGAAACGGCAGACTGTGAGGTGTCTCGCTGACTCCCTCTTCGGAGGAGAAGACGCGAGTCCCTTTAAATGTACGTTCGCTCTTTCTTGTGTTTAAGTAGAAGTGTGACTTTCGAACGCAGTGATCTGTTTGGATCGCTTTGCTCGAGTAGGCGACTTCGGTTAGGACATTAAAGGAAGCAACCCTATTGGCGGTATGTTAGGCTTCGGCCCGACTTTGCAGCTGACGGTGTGTTGTTTTCTGTTCTTTCCTTGAGGTGTACCTGTCTTGTGTGAGGCAATGGTCTaGGCAAATGGTTATTGTGTAGTAGGAAGTTGCTGCTCTTGAACGCCCTGTttTCGGATAGGGTAAAGGAGGCAACACCAATTTGGGATAGTCTTTGATTTATCATTGGCGCTCTTTCTAATTGGACCTGATATCAGGCAAGACTACCCGCTGAACTTAAGCATATTAATAAGCGGAGGAAAAGAAACTAACAAGGATTCCCCTAGTAACGGCGAGTGAAGCGGGATGAGCTCAAGCTTAAAATCTCTGTGCCAGTTTGGCATGGCGAATTGTAGTCTATGGAGGCGCTATCAGTGCGATTGTTCGGGG.

Bacteriological PCR analysis of the affected skin samples (taken on day 34) were positive for *Vibrio parahaemolyticus*, considered a bacterial commensal of harbour porpoise’s skin flora. Bacteriological cultures (of samples taken during autopsy on day 105) were unremarkable, with no growth obtained after 14 days of culture in all sampled sites, except for a few *Pseudomonas* sp. from the tracheobronchial lymph nodes and also from skin, along with a few *Acinetobacter* sp.

Virological PCR analysis on samples from brain, lung, kidney and urinary bladder collected during autopsy on day 105 tested negative for the presence of morbilliviral RNA.

## Discussion

Previously, pythiosis has been reported in a limited number of humans and other terrestrial mammals and a bird, with typically *Pythium insidiosum* as the causative pathogen [3]. Here, we diagnosed *P. flevoense* infection as the cause of dermatitis in a harbour porpoise. To our knowledge, it is the first time both for the diagnosis of pythiosis in a marine mammal and for *P. flevoense* as a cause of disease in a mammalian species. *P. flevoense* infections were reported previously in non-mammalian species only; ayu fish larvae [23] and freshwater copepods [22]. Evidence from affected skin samples that pointed to a *Pythium* species as the causative pathogen was indicated by histopathological evaluation and confirmed by DNA sequencing that identified this particular, pathogenic species. Histological examination, especially in silver stains, revealed intralesional presence of fungus-like hyphae of approximately 7–10 μm diameter with rare septations and irregular branching (Figure 2) matching the general morphology of *Pythium* species [3, 15]. The colocalization of the *Pythium* hyphae with the skin lesions, combined with the absence of any other pathogens, supports *P. flevoense* as the cause of this chronic active erosive dermatitis. The genetic analysis conclusively matched a known sequence of *Pythium flevoense*, which was isolated and characterized for the first time from a soil sample from Flevoland in the Netherlands [21]. The species identification in our case pointed towards *P. pectinolyticum* [28] initially but was subsequently identified as *P. flevoense*. This illustrates the importance of scrutinizing differentiation in closely related *Pythium* spp. and as ITS is defined as the barcode region for fungi [19], additional distinguishing molecular markers may be necessary for future studies.

Another unusual aspect of this case was the temperate saltwater environment, which is in contrast with the subtropical stagnant freshwater conditions, such as swamps and rice fields, where infections with *P. insidiosum* in mammals have been reported until now [3, 11, 29, 30]. Similarly, *P. flevoense* infections in a crustacean (copepod, *Parabroteas sarci*) were detected increasingly at warmer freshwater temperatures >20 °C in Argentina [22]. Nonetheless, oomycetes have been reported to occur in marine and terrestrial environments also, and *Pythium* species have been isolated from seaweeds and algae in temperate coastal saltwater environments [31, 32]. Mortality and systemic *P. flevoense* infection was reported in ayu fish larvae reared in artificial saltwater conditions at 12–15 °C in a fishery in Japan [23]. Even so, pythiosis has not been reported to occur in either temperate or saltwater environments in marine mammals or other mammalian species.

As pythiosis is not known to be contagious between mammals, infection in our case most likely occurred at sea or in the marine centre, presumably from infectious zoospores of *P. flevoense* in the water. Skin wounds in animals and humans are reported to serve as potential port d’entrée for the infectious motile zoospores [2]. In our case the porpoise was found trapped bycaught in a pound net, unable to free itself. Such entanglements in fishing nets are known to potentially inflict skin wounds on harbour porpoises [33, 34]. Furthermore, as seaweeds and algae typically encroach nets and weirs, they may also harbour oomycetes [31, 32]. Therefore, it is not unlikely that this event may have injured the porpoise’s skin, permitting infection. In support to this reasoning were the several skin scars on the left side on the body found during autopsy. Whether these older intact scars actually resulted from this entanglement and served as a potential infection route is not known. Nor was it substantiated that local seaweeds and/or algae carried oomycetes, or that zoospores were ubiquitously present in these salt waters. As *P. flevoense* was isolated from soil samples previously, another possible explanation is that the seawater in the bay and basin may have been contaminated via freshwater runoff from land. However, another harbour porpoise that was kept in a separated adjacent basin circulated with the same seawater remained healthy without any signs of dermatitis or skin lesions.

Immunosuppression and hemopathies are risk factors for contracting pythiosis. For example, there are scientific reports regarding pythiosis with haemoglobinopathies, anaemia and leukaemia as risk factors for infection in humans [9–12], and chemically immunosuppressed mice showed higher susceptibility to induced subcutaneous pythiosis [35]. Environmental toxic pollutants like PCBs accumulated in adipose tissues released during periods of reduced food intake may cause immunosuppression in marine mammals [36]. Although no toxicological analysis was performed in this case, there was neither haematological evidence for leukopenia and anaemia nor was there histopathological evidence of lymphoid depletion in spleen and lymph nodes to indicate immunosuppression. Additionally, this porpoise was in good physical condition with a substantial blubber thickness without signs of cachexia or lipolysis, and showed no haematological abnormalities upon acceptance in the centre. Possible morbillivirus-induced immunosuppression [37] was excluded based on the lack of histological morbilliviral inclusion bodies in combination with negative morbilliviral-specific PCR analysis of brain, lung, kidney and urinary bladder. Pregnancy, a possible risk factor for contracting pythiosis in horses [13], was not present in this young female porpoise.

Initial clinical examination and diagnostics implicated a fungal organism as a cause of disease in this case, although a specific identification at that time was not made. Marine mammals are known to be susceptible to fungal diseases. In particular, respiratory aspergillosis has been reported in harbour porpoises [25], harbour seals (*Phoca vitulina*) and several species of cetaceans [38, 39]. Rare cases of disseminated fungal infections are reported in harbour porpoises as well, with *Rhizopus* sp. [40], *Cryptococcus gatti* [41] and *Candida albicans* [42] as causative organisms.

Despite various antifungal and antibiotic treatments in this case, the clinical course subsequently developed into a progressive severe dermatitis. Retrospectively, such a clinical course, with difficulties in initial diagnosis and resistance to antifungal therapy, is typical for pythiosis, which is not a true fungal disease [3, 43, 44]. The reported treatment of choice concerns radical surgery to remove all infected tissues, in conjunction with antibiotic treatments and/or immunotherapies, but in general the prognosis is poor and disseminated cases usually progress to mortality [2, 3].

In conclusion, the diagnosis of *P. flevoense* as cause of this dermatitis was evidenced by its intralesional presence in combination with histological absence and microbiological exclusion of other potential pathogens, such as bacteria, true fungi, protozoa, and viruses. Also, the possibility of this particular *Pythium* species being an opportunistic infection was considered and excluded because: (1) there was no evidence from haematology, histopathology, or (molecular) microbiology to suggest underlying immunosuppression, and (2) infection occurred in a non-pregnant initially healthy young female harbour porpoise in good physical condition without co-morbidities. Furthermore, our findings of this new case of pythiosis in a harbour porpoise are consistent with observations from previous investigators that the host range of pythiosis is increasing [2, 3, 5]. So given our diagnosis of cutaneous pythiosis caused by this pathogenic *Pythium* species, we recommend including *P. flevoense* as a differential diagnosis for dermatitis in marine mammals, and more specifically, in harbour porpoises that have been entangled in fishing nets.

### Supplementary Information

**Additional file 1. Results of blood analyses for haematologic and biochemistry parameters from harbour porpoise Idun.** Reference intervals from free-ranging harbour porpoises from Danish waters with a healthy clinical appearance (except for the intervals marked with an *; these originated from healthy long-term captive harbour porpoises) according to Siebert et al. [27]. The lower and higher thresholds of these intervals represent the 10^th^ and 90^th^ percentiles for each blood parameter with their 95% bootstrapped confidence intervals, respectively. NA: not available.

## Data Availability

The datasets supporting the conclusions of this article are included within the article (and its additional file).
