# Supplementary material for: Infection with Pythium flevoense in a harbour porpoise (Phocoena phocoena) as a novel cause of dermatitis in marine mammals
Source: Vet Res. 2023 Nov 2;54:102. doi: 10.1186/s13567-023-01226-1 (PMC10623814; doi:10.1186/s13567-023-01226-1)
Supplement: Supplementary file 1 — Additional file 1. Results of blood analyses for haematologic and biochemistry parameters from harbour porpoise Idun. Reference intervals from free-ranging harbour porpoises from Danish waters with a healthy clinical appearance (except for the intervals marked with an *; these originated from healthy long-term captive harbour porpoises) according to Siebert et al. [27]. The lower and higher thresholds of these intervals represent the 10th and 90th percentiles for each blood parameter with their 95% bootstrapped confidence intervals, respectively. NA: not available. [file 13567_2023_1226_MOESM1_ESM.docx]

**Additional file 1. Haematologic and biochemistry parameters.**

| **Date** | | **Day 0** | **Day 3** | **Day 5** | **Day 7** | **Day 10** | **Day 15** | **Day 17** | **Day 20** | **Day 25** | **Day 29** | **Day 34** | **Day 43** | **Day 48** | **Day 51** | **Day 57** | **Day 62** | **Day 90** | **Day 104** | **Reference interval [27]** |
| --- | --- | --- | --- | --- | --- | --- | --- | --- | --- | --- | --- | --- | --- | --- | --- | --- | --- | --- | --- | --- |
| **Parameter** | **Unit** |  |  |  |  |  |  |  |  |  |  |  |  |  |  |  |  |  |  |  |
| **Red blood cells** | **RBC (T/l)** | **5.26** | **5.49** | **5.60** | **5.59** | **5.62** | **5.48** | **5.49** | **5.62** | **5.62** | **5.73** | **5.90** | **5.66** | **5.11** | **5.04** | **5.03** | **4.90** | **4.72** | **4.08** | **4.26 – 6.59** |
| **Haemoglobin** | **HGB (g/dl)** | **17.2** | **17.7** | **18.1** | **18.1** | **18.2** | **18.0** | **17.8** | **8.7** | **18.1** | **18.5** | **18.8** | **17.9** | **16.3** | **16.5** | **15.8** | **15.4** | **13.6** | **11.1** | **13.04 – 19.34** |
| **Haematocrit** | **HCT (%)** | **45.8** | **47.9** | **48.9** | **49.2** | **49.4** | **47.4** | **47.0** | **48.0** | **48.5** | **49.4** | **51.0** | **48.6** | **43.2** | **43.1** | **42.5** | **41.6** | **37.0** | **31.2** | **37.0 – 57.0** |
| **Mean cell volume** | **MCV (fl)** | **87.1** | **87.2** | **87.3** | **88.0** | **87.9** | **86.5** | **85.6** | **85.4** | **86.3** | **86.2** | **86.4** | **85.9** | **84.5** | **85.5** | **84.5** | **84.9** | **78.4** | **76.5** | **79.60 – 92.00** |
| **Mean corpuscular HGB concentrate** | **MCHC (g/dl)** | **37.6** | **37.0** | **37.0** | **36.8** | **36.8** | **38.0** | **37.9** | **39.0** | **37.3** | **37.4** | **36.9** | **36.8** | **37.7** | **38.3** | **37.2** | **37.0** | **36.8** | **35.6** | **31.00 – 36.08** |
| **Platelets** | **PLT (G/l)** | **188.3** | **157.0** | **205.0** | **184.5** | **176.5** | **134.8** | **120.8** | **119.1** | **138.8** | **140.4** | **129.2** | **135.8** | **160.5** | **168.3** | **217.3** | **219.5** | **209.6** | **219.9** | **118.60 – 326.80** |
| **Mean platelet volume** | **MPV (fl)** | **12.0** | **12.0** | **12.3** | **12.6** | **12.8** | **12.5** | **12.9** | **12.8** | **13.3** | **13.3** | **13.4** | **13.8** | **13.8** | **14.0** | **14.3** | **14.6** | *NA* | *NA* | **9.08 – 14.60** |
| **Eosinophils** | **EOS (%)** | **16.0** | **17.0** | **19.2** | **16.1** | **16.4** | **11.0** | **9.5** | **8.6** | **9.4** | **5.8** | **5.4** | **3.8** | **2.6** | **3.9** | **5.3** | **3.7** | **2.2** | **32.0** | **6.00 – 23.94** |
| **White blood cells** | **WBC (G/l)** | **5.88** | **10.22** | **9.48** | **8.34** | **7.27** | **9.35** | **9.72** | **10.73** | **11.60** | **9.48** | **10.62** | **17.77** | **18.05** | **19.86** | **21.13** | **20.16** | **22.26** | **28.22** | **3.56 – 7.94** |
| **Lymphocytes %** | **LYM (%)** | **45.2** | **49.4** | **54.0** | **58.0** | **53.0** | **42.4** | **41.2** | **39.6** | **28.0** | **28.3** | **26.3** | **24.6** | **18.5** | **28.9** | **20.8** | **16.9** | **16.3** | **11.1** | **9.20 – 56.80** |
| **Monocytes %** | **MONO (%)** | **4.1** | **5.7** | **5.7** | **5.3** | **5.9** | **5.7** | **6.5** | **7.7** | **7.4** | **6.3** | **6.1** | **6.4** | **9.5** | **11.2** | **6.4** | **6.2** | **6.6** | **6.0** | **0.00 – 7.60** |
| **Neutrophils %** | **NEU (%)** | **34.5** | **27.7** | **20.9** | **20.5** | **24.6** | **40.5** | **42.7** | **43.8** | **0.7** | **59.3** | **61.9** | **64.9** | **69.1** | **55.6** | **67.3** | **72.7** | **74.5** | **50.6** | *NA* |
| **Basophils %** | **BASO (%)** | **0.2** | **0.2** | **0.2** | **0.1** | **0.1** | **0.4** | **0.1** | **0.3** | **0.2** | **0.3** | **0.3** | **0.3** | **0.3** | **0.4** | **0.2** | **0.5** | **0.4** | **0.3** | *NA* |
| **Absolute lymphocyte count** | **ALC (G/l)** | **2.66** | **5.05** | **5.12** | **4.84** | **3.85** | **3.96** | **4.00** | **4.25** | **3.30** | **2.68** | **2.79** | **4.38** | **3.34** | **5.74** | **4.40** | **3.41** | **3.62** | **3.14** | **0.84 – 3.28** |
| **Absolute monocyte count** | **AMC (G/l)** | **0.24** | **0.58** | **0.54** | **0.44** | **0.43** | **0.53** | **0.63** | **0.83** | **0.86** | **0.60** | **0.65** | **1.13** | **1.71** | **2.23** | **1.35** | **1.25** | **1.46** | **1.69** | **0.00 – 0.20** |
| **Absolute neutrophil count** | **(G/l)** | **2.03** | **2.83** | **1.98** | **1.71** | **1.79** | **3.79** | **4.16** | **4.70** | **6.34** | **5.62** | **6.58** | **11.53** | **12.48** | **11.05** | **14.22** | **14.66** | **16.58** | **14.29** | *NA* |
| **Absolute eosinophil count** | **(G/l)** | **0.94** | **1.74** | **1.82** | **1.34** | **1.19** | **1.03** | **0.92** | **0.92** | **1.08** | **0.55** | **0.57** | **0.68** | **0.47** | **0.77** | **1.11** | **0.74** | **0.50** | **9.02** | *NA* |
| **Absolute basophil count** | **(G/l)** | **0.01** | **0.02** | **0.02** | **0.01** | **0.10** | **0.04** | **0.01** | **0.03** | **0.02** | **0.03** | **0.03** | **0.05** | **0.05** | **0.07** | **0.05** | **0.10** | **0.10** | **0.30** | *NA* |
| **Absolute granulocyte count** | **AGC (G/l)** | *NA* | *NA* | *NA* | *NA* | *NA* | *NA* | *NA* | *NA* | *NA* | *NA* | *NA* | *NA* | *NA* | *NA* | *NA* | *NA* | *NA* | *NA* | **1.74 – 6.22** |
|  |  |  |  |  |  |  |  |  |  |  |  |  |  |  |  |  |  |  |  |  |
| **Calcium** | **Ca (mmol/l)** | **2.28** | **2.26** | **2.29** | **2.26** | **2.30** | **2.22** | **2.15** | **2.15** | **2.28** | **2.22** | **2.19** | **2.03** | **2.15** | **2.20** | **2.19** | **2.13** | **2.33** | **2.28** | **2.01 – 2.63** |
| **Iron** | **Fe (µmol/l)** | *NA* | **66** | **58** | **58** | **56** | *NA* | *NA* | *NA* | *NA* | *NA* | *NA* | *NA* | *NA* | *NA* | **35** | **23** | *NA* | *NA* | **11.92 – 50.04** |
| **Chlorine** | **Cl (mmol/l)** | **117** | **119** | **122** | **121** | **121** | **118** | **117** | **117** | **116** | **121** | **121** | **122** | **121** | **121** | **124** | **121** | **119** | **121** | **88.20 – 129.50*** |
| **Potassium** | **K (mmol/l)** | **3.7** | **3.7** | **3.6** | **3.6** | **4.0** | **4.1** | **3.8** | **3.8** | **3.7** | **3.3** | **3.2** | **3.6** | **3.7** | **3.4** | **4.8** | **4.3** | **4.0** | **3.6** | **3.35 – 4.96** |
| **Sodium** | **Na (mmol/l)** | **154** | **161** | **158** | **161** | **157** | **157** | **159** | **158** | **157** | **159** | **161** | **157** | **157** | **160** | **160** | **159** | **152** | **154** | **146.50 – 157.90** |
| **Phosphorus** | **PHOS (mmol/l)** | **0.81** | **2.16** | **2.07** | **2.27** | **2.20** | **1.61** | **1.89** | **2.05** | **1.83** | **1.59** | **1.62** | **2.33** | **2.58** | **2.37** | **3.34** | **3.06** | **3.19** | **2.29** | **0.92 – 3.44** |
| **Gamma glutamyl transferase** | **GGT (U/l)** | **39** | **49** | **44** | **46** | **56** | **55** | **60** | **69** | **92** | **90** | **87** | **57** | **67** | **107** | **94** | **79** | **67** | **77** | **7.30 – 34.40** |
| **Alkaline phophatase** | **ALP (U/l)** | **764** | **497** | **470** | **512** | **563** | **627** | **601** | **664** | **464** | **419** | **388** | **333** | **288** | **292** | **260** | **267** | **231** | **308** | **56.00 – 751.60** |
| **Aspartate aminotransferase** | **AST (U/l)** | **223** | **309** | **47** | **314** | **367** | **474** | **467** | **546** | **475** | **419** | **484** | *NA* | *NA* | *NA* | **464** | **419** | *NA* | **773** | **261.00 – 432.40** |
| **Alanine aminotransferase** | **ALT (U/l)** | **33** | **50** | **47** | **76** | **99** | **106** | **137** | **113** | **106** | **107** | **133** | **112** | **263** | **248** | **133** | **100** | **72** | **244** | **73.20 – 134.60** |
| **Lactic acid dehydrogenase** | **LDH (U/l)** | **1403** | **1668** | **1703** | **1763** | **1757** | **2129** | **2224** | **2509** | **2312** | **1902** | **1717** | **1808** | **2461** | **2426** | **2138** | **1950** | **1638** | *NA* | **440.00 – 765.00** |
| **Cholesterol** | **Chol (mmol/l)** | **2.04** | **3.11** | **3.22** | **3.41** | **3.44** | **2.99** | **2.98** | **3.00** | **3.46** | **4.10** | **3.82** | **3.20** | **3.80** | **4.57** | **4.31** | **4.01** | **4.38** | **2.81** | **2.84 – 5.25** |
| **Urea** | **UREA (mmol/l)** | **9.1** | **12.1** | **13.0** | **15.9** | **13.9** | **10.3** | **10.4** | **11.8** | **18.6** | **17.0** | **12.8** | **13.2** | **15.0** | **15.6** | **15.6** | **12.2** | **11.6** | **11.6** | **9.89 – 16.80** |
| **Creatinine** | **CREA (µmol/l)** | **64** | **51** | **52** | **39** | **42** | **42** | **40** | **38** | **36** | **28** | **27** | **36** | **33** | **29** | **36** | *NA* | **35** | **22** | **59.23 – 119.34** |
| **BUN/CREA** |  | **36** | **57** | **60** | *NA* | **78** | **58** | **58** | **83** | *NA* | **160** | **120** | **93** | **105** | **147** | *NA* | *NA* | **80** | **160** | *NA* |
| **Albumin** | **ALB (g/l)** | **30** | **31** | **32** | **33** | **36** | **32** | **33** | **32** | **33** | **34** | **34** | **32** | **32** | **31** | **33** | **33** | **37** | **31** | **27.80 – 37.00*** |
| **Globuline** | **GLOB (g/l)** | **36** | **38** | **36** | **38** | **39** | **36** | **31** | **37** | **37** | **38** | **42** | **41** | **39** | **42** | **46** | **44** | **59** | **60** | **34.80 – 51.00*** |
| **Total protein** | **TP (g/l)** | **66** | **69** | **68** | **71** | **74** | **68** | **64** | **69** | **70** | **72** | **76** | **73** | **71** | **73** | **79** | **77** | **96** | **91** | **63.47 - 86.62** |
